# Ubiquitin signals the demise of AMPA receptors

**DOI:** 10.18632/oncotarget.4645

**Published:** 2015-06-25

**Authors:** Jocelyn Widagdo, Victor Anggono

**Affiliations:** Clem Jones Centre for Ageing Dementia Research, Queensland Brain Institute, The University of Queensland, Brisbane, Australia

Efficient communication between neurons is essential for all activities ranging from muscle movement and motor coordination to higher-order information processing and cognitive functions such as learning and memory. The majority of fast excitatory synaptic transmission in the mammalian central nervous system is mediated by the release of glutamates from the presynaptic terminal and their binding to the ionotropic AMPA-type glutamate receptors (AMPARs) on the postsynaptic membrane. Dynamic changes in the trafficking and expression of synaptic AMPARs are crucial for synaptic plasticity. Therefore, aberrant trafficking of AMPARs often leads to deficits in learning and memory [1]. More importantly, dysregulation of AMPAR function has been implicated in various brain disorders, including addiction and, neuropsychiatric and neurodegenerative diseases. Elucidating the mechanisms that regulate AMPAR trafficking and turnover is therefore critical to understanding the fundamental biological process of normal brain function, as well as providing insights for the identification of potential therapeutic targets.

Ubiquitination, a reversible post-translational modification that is already known to play important roles in activity-dependent remodelling of the postsynaptic density, synaptic plasticity, learning and memory, has recently been shown to directly target AMPAR subunits in mammalian central neurons [2, 3]. However, details regarding which AMPAR subunits are affected, as well as where and how ubiquitination affects receptor functions, have proven inconsistent between studies. These controversies were addressed in our recent systematic study [4]. We found that all AMPAR subunits (GluA1-4) undergo activity-dependent ubiquitination when neurons are stimulated with the agonist AMPA or the GABA_A_ receptor antagonist, bicuculline. This process is dependent on calcium (Ca^2+^) influx through the L-type voltage-gated Ca^2+^ channels, which in turn activates the downstream effector Ca^2+^/calmodulin-dependent kinase II (CaMKII). How CaMKII influences AMPAR ubiquitination is currently unknown, but it is plausible that it directly phosphorylates downstream E3 ligases, such as Nedd4-1 or RNF167, and activates their ubiquitin ligase activity. Indeed, such a mechanism has been demonstrated for fibroblast growth factor-induced activation of Nedd4-1 ubiquitin ligase activity through the phosphorylation of a tyrosine residue in the HECT domain by the tyrosine kinase c-Src [5].

To demonstrate the direct role of ubiquitination on AMPAR function, we performed a systematic mapping of the sites of ubiquitination on the GluA1 and GluA2 subunits of AMPARs. We identified Lys-868 and Lys-870/Lys-882 in the carboxyl-terminal domains as the major ubiquitination sites, mutations of which account for more than 80% reduction in the levels of GluA1 and GluA2 ubiquitination, respectively. These Lys-Arg mutants allowed us to probe the effects of ubiquitination on AMPAR functions, including surface expression, endocytosis, intracellular trafficking and turnover, without manipulating the expression levels of E3 ligases, which target many other protein substrates in neurons. In contrast to previous studies [3, 6], we found no evidence of a role of ubiquitination in regulating the surface expression of AMPARs under basal conditions. This finding is consistent with the fact that the levels of AMPAR ubiquitination are extremely low in the absence of neuronal activity [2-4].

One surprising result emerged from our study is that the GluA1 and GluA2 ubiquitin-deficient mutants display normal internalisation following AMPA stimulation, which contradicts previously reported data [3, 6]. Our findings can be explained by the fact that activity-induced (both by AMPA and bicuculline) ubiquitination of AMPARs is completely abolished by two independent endocytosis inhibitors, dynasore and dynole, suggesting that ubiquitination of AMPARs must occur post-endocytosis [2, 4]. Moreover, we also found that the GluA1 and GluA2 subunits are modified by K63-linked polyubiquitination [4], which predominantly regulates intracellular sorting of transmembrane receptors to the late endosome/lysosomal compartments. Indeed, our data demonstrated that the GluA1 ubiquitin-deficient mutants escape the lysosomal degradation pathway, and are instead recycled back to the plasma membrane. As a consequence, these mutants are more resistant to agonist-induced degradation.

Although the role of direct ubiquitination of AMPAR subunits in receptor endocytosis remains controversial, there can be no doubt that the role of AMPAR ubiquitination in synaptic plasticity, learning and memory is the next outstanding question that needs to be addressed. A recent study has implicated the role of AMPAR ubiquitination in homeostatic synaptic scaling [7]; however whether or not this chemical modification is essential for long-term potentiation and/or long-term depression is unknown. GluA1 and GluA2 ubiquitin-deficient knock-in mice could hold the answer. In addition, primary neurons derived from these mice would allow us to better study the physiological role of activity-induced AMPAR ubiquitination without the necessity of overexpressing exogenous AMPAR mutants, which may potentially be a confounding factor. Given the tight association between the ubiquitin-proteasome pathway with many neurodegenerative and neuropsychiatric disorders, and the fundamental importance of AMPARs in normal brain function, we are hopeful that understanding the regulatory mechanisms underlying AMPAR function through protein ubiquitination will not only shed light on the molecular basis of learning and memory, but also provide insight into the principles underpinning disease aetiology.
